# Agonists and Inhibitors of the cGAS-STING Pathway

**DOI:** 10.3390/molecules29133121

**Published:** 2024-06-30

**Authors:** Xiaoxuan Yu, Linxiang Cai, Jingyue Yao, Cenming Li, Xiaoyong Wang

**Affiliations:** 1 Jiangsu Collaborative Innovation Center of Chinese Medicinal Resources Industrialization, School of Medicine, Nanjing University of Chinese Medicine, Nanjing 210023, China; 2 State Key Laboratory of Pharmaceutical Biotechnology, School of Life Sciences, Nanjing University, Nanjing 210023, China; 3 State Key Laboratory of Coordination Chemistry, School of Chemistry and Chemical Engineering, Nanjing University, Nanjing 210023, China; 4 Department of Pharmacy, Fourth Military Medical University, Xi’an 710032, China; yaojingyue1114@163.com

**Keywords:** cGAS-STING, agonist, inhibitor, drug design, immunotherapy

## Abstract

The cyclic GMP-AMP synthase (cGAS)-stimulator of interferon genes (STING) pathway is pivotal in immunotherapy. Several agonists and inhibitors of the cGAS-STING pathway have been developed and evaluated for the treatment of various diseases. The agonists aim to activate STING, with cyclic dinucleotides (CDNs) being the most common, while the inhibitors aim to block the enzymatic activity or DNA binding ability of cGAS. Meanwhile, non-CDN compounds and cGAS agonists are also gaining attention. The omnipresence of the cGAS-STING pathway in vivo indicates that its overactivation could lead to undesired inflammatory responses and autoimmune diseases, which underscores the necessity of developing both agonists and inhibitors of the cGAS-STING pathway. This review describes the molecular traits and roles of the cGAS-STING pathway and summarizes the development of cGAS-STING agonists and inhibitors. The information is supposed to be conducive to the design of novel drugs for targeting the cGAS-STING pathway.

## 1. Introduction

**Figure 1:**
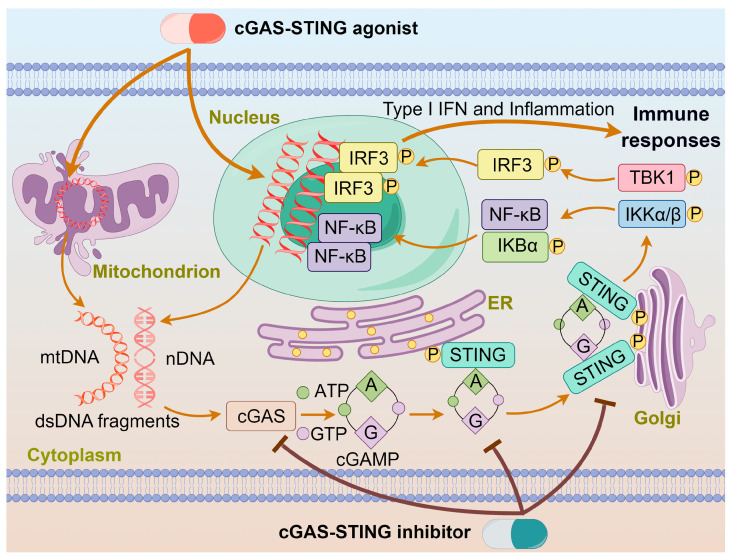
The action of agonist or inhibitor on the cGAS-STING pathway. dsDNA binds to cGAS and activates cGAS, leading to the synthesis of cGAMP. cGAMP binds to the STING located on the ER, inducing conformational changes and oligomerization of STING. The STING oligomers release from the ER and translocate to the Golgi apparatus, where they recruit TBK1 and IKK kinases. Phosphorylated IRF3 and IκBα form functional signalosomes and translocate into the nucleus to induce the expression of IFNs and immune-stimulated genes. The cGAS-STING agonist or inhibitor promotes or impedes these processes.

Chemotherapy, surgery, and radiation therapy are three major treatments for cancers. In recent years, immunotherapy is emerging as another treatment for cancers. Generally, chemotherapy eliminates cancers by direct cytotoxic effects; however, it often encounters drug resistance and toxic side effects. By contrast, immunotherapy aims to regulate immunological responses through activating the immune defense system or reshaping the tumor microenvironment (TME). Many chemotherapeutics can cause cellular DNA damages, which may initiate innate immunity to kill cancer cells. Cyclic GMP-AMP synthase (cGAS) is an intracellular detector in the innate immune system that recognizes double-stranded DNA (dsDNA). It converts ATP and GTP into a signaling molecule cyclic GMP-AMP (cGAMP), which then serves as a second messenger to activate the stimulator of interferon genes (STING) protein and trigger the production of interferons in immune responses [1,2]. Mammalian cellular DNA is mainly localized within the nucleus or mitochondria, with only a minimal amount presenting in the cytoplasm, which keeps cGAS in an inactive state [3,4]. During drug treatment, cGAS is activated in a sequence-independent manner upon binding to abnormal dsDNA to produce cGAMP and transmit signals from upstream cells to downstream adaptor proteins. It binds to the C-terminus of the STING protein, facilitating its transport from the endoplasmic reticulum (ER) to the Golgi apparatus by high-order oligomerization and tetramer formation [5]. Once localized to the Golgi apparatus, the STING protein undergoes post-translational palmitoylation at two cysteine residues (Cys88/Cys91) in the N-terminal domain, inducing conformational changes and oligomerization [3]. The STING is primarily expressed in the ER and translocates to the Golgi apparatus upon activation, recruiting TANK-binding kinase 1 (TBK1) and IκB kinase (IKK), which undergo autophosphorylation [6]. The phosphorylated TBK1 and IKK stimulate the transcription factors interferon nuclear factor κB (NF-κB) and interferon regulatory factor 3 (IRF3), eliciting the expression of proinflammatory cytokines such as interleukin 6 (IL-6) and type I interferons (IFN-I) to kill cancer cells [7,8]. The action of an agonist or inhibitor on the cGAS-STING pathway is schematized in Figure 1.

Immune evasion is one of the key features of tumor. Activating antitumor immunity is an effective method against tumors [9]. Activation of the cGAS-STING pathway triggers a strong antitumor immune response by inducing a robust production of IFN-I, which is crucial for activating immune cells, particularly CD8^+^ T cells [10,11]. As main effector cells for tumor immune surveillance and clearance, CD8^+^ T cells recognize tumor antigens presented by antigen-presenting cells (APCs) such as dendritic cells (DCs) through the major histocompatibility complex class I (MHC-I), leading to the killing of cancer cells [10]. In APCs, particularly in tumor-resident DCs, the activation of the cGAS-STING pathway lays the basis for the activation and proliferation of T cells, leading to the infiltration of CD8^+^ T cells and the initiation of immune responses against tumors [12]. Additionally, IFN-I reduces the acidification rate of endo-lysosomes in DCs, prolonging the storage and binding time of tumor antigens and MHC-I molecules in the endosomal compartments, assisting DCs in antigen presentation to CD8^+^ T cells [13]. T cell infiltration is a hallmark of IFN-I production and is associated with a positive overall prognosis of tumor patients. It has been demonstrated that after immunogenic tumor transplantation into syngeneic mice, STING-deficient mice exhibited faster tumor growth compared to the wild-type mice [14].

In the TME, activation of the cGAS-STING pathway mediates the activity of immune cells, including the maturation of DCs, cross-priming of tumor-related antigens, and cytotoxic T-lymphocytes [15,16]. Natural killer (NK) cells, as a type of cytotoxic lymphocyte, recognize tumor cells lacking MHC class molecules [17]. The cGAS-STING pathway can enhance the antitumor immune surveillance and clearance functions mediated by NK cells. The tumor-derived 2′3′-cGAMP promotes the expression of T cell transcription factor 1 (TCF-1) in NK cells, which are characterized by immune memory features, thereby expanding the number of TCF-1^+^ NK cells to enhance the tumor-killing ability. Furthermore, 2′3′-cGAMP induces IFN-I to upregulate IL-15 expression in the TME, leading to the increase of IL-15 receptors on DCs and NK cells, and hence enhancing the antitumor activity of NK cells [18,19]. Activation of the cGAS-STING pathway in tumors may induce the expression of immune checkpoint molecules, suppressing the recognition and killing of tumor cells by immune ones [20]. cGAS is essential for the antitumor effect of the immune checkpoint blockade [21], and STING agonist-formulated vaccines can cure tumors resistant to the PD-1 blockade [22]. STING agonists induce the expression of PD-L1 on the surface of tumor cells, thereby inhibiting specific killing of tumor antigens, providing a rationale for the combined use of STING agonists with PD-1/PD-L1 antibodies in tumor immunotherapy [21,22]. The above findings signify that the cGAS-STING pathway plays a vital role in antitumor immunity.

On the other hand, STING also shows a pro-tumorigenic effect in some cases [23]. A pan-cancer analysis revealed that high activation of the cGAS-STING pathway and its downstream signaling components such as TBK1 and IRF3 is associated with reduced immune cell infiltration in the TME and poor prognosis for colorectal and gastric adenocarcinomas. Furthermore, an analysis on The Cancer Genome Atlas (TCGA) database also showed that the expression of STING is positively correlated with the infiltration of almost all immune cells, including myeloid-derived suppressor cells (MDSCs) and regulatory T cells (Tregs) [24]. For example, the chromosomal instability in tumor cells can lead to sustained activation of the cGAS-STING pathway, inducing chronic inflammation and immunosuppressive TME, desensitizing cells to inflammatory responses and aiding tumor cells in immune escape [24,25]. Mutagenic agents such as 7,12-dimethyl-benz(a)anthracene (DMBA), cisplatin, and etoposide promote skin carcinogenesis by triggering the STING-dependent production of inflammatory cytokines and the infiltration of phagocytic cells [26]. Similarly, chromosomally unstable tumor cells exploit the persistent activation of noncanonical NF-κB signaling following STING activation to boost their invasion and metastasis [27]. One hallmark of the immunosuppressive TME is the increased expression of immune checkpoint protein indoleamine 2,3-dioxygenase (IDO). STING activation can stimulate IDO activity in DCs within the TME, which in turn activates Tregs, suppresses the infiltration of CD8^+^ T cells into tumors, and dampens their antitumor activity [28].

As a crucial component of the innate immune system, the cGAS-STING pathway plays a significant regulatory role in the occurrence and development of tumors [29]. It is involved in the inhibition of tumor progression through promoting the cytotoxic effects of T cells and NK cells, inducing apoptosis and autophagy [30,31]. Meanwhile, it promotes tumor development through chronic inflammation induced by its sustained activation and inhibition on DNA damage repair [10]. This review will provide a concise summary on the recent advances in agonists and inhibitors of the cGAS-STING pathway. We hope it is of value to biomedical researchers working in the domain of drug design and clinical translation.

## 2. cGAS-STING Agonists

The cGAS-STING pathway bridges innate and adaptive immunity against tumors [32,33]. Both indirect and direct activations of the cGAS-STING pathway can stimulate antitumor immune responses. Indirect agonists do not bind directly to STING but instead induce a series of reactions that activate the cGAS-STING pathway. Indirect activation includes various cancer treatments, such as DNA-damaging chemotherapies using cisplatin [34], camptothecin [35], doxorubicin [36], paclitaxel [37], and etoposide [38,39]. Some activation is induced by impairing the DNA damage response using poly ADP-ribose polymerase (PARP) [40,41], ataxia telangiectasia and rad3-related (ATR) inhibitors [42], as well as radiation [43]. The activation within tumor cells is triggered by cellular dsDNA accessible to cGAS. Direct agonists bind directly to STING. Direct activation supports the formation of memory T cells and contributes to long-lasting immune responses against cancer [32].

**Figure 2:**
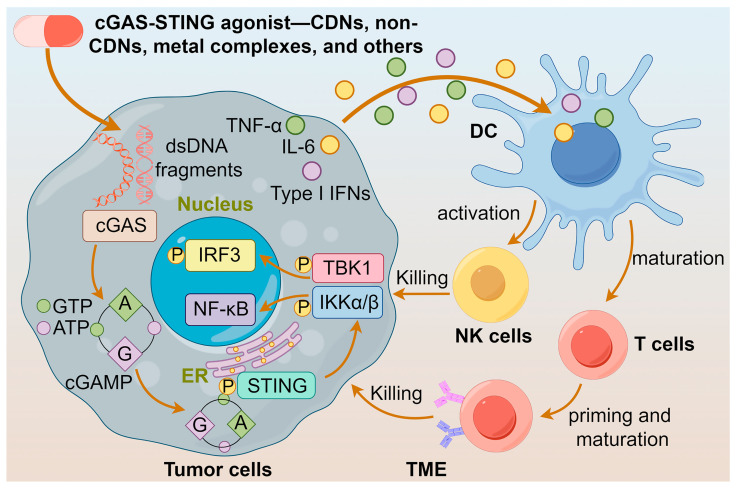
Actions of different cGAS-STING agonists. cGAS-STING agonists enhance the release of IFNs and maturation of DCs by activating the cGAS-STING pathway, leading to the activation of T cells and NK cells, and ultimately to the killing of tumor cells.

Furthermore, cGAS-STING agonists stimulate the secretion of the chemokine CXCL10 in an IFN-I-dependent manner [44], which facilitates the recruitment of antigen-specific CD8^+^ T cells to infiltrate into the tumor and elicit an effective antitumor immune response [33]. Notably, when the STING protein is activated in tumor cells, it triggers the production of IFN-β, which reverses the downregulation of MHC-I expression, leading to the escape of tumor cells from immune recognition by cytotoxic T-lymphocytes (CTLs) [45]. The multifaceted effects of cGAS-STING agonists suggest that they are potential anticancer agents. The actions of diversified agonists are summarized in Figure 2.

### 2.1. Cyclic Dinucleotides

**Figure 3:**
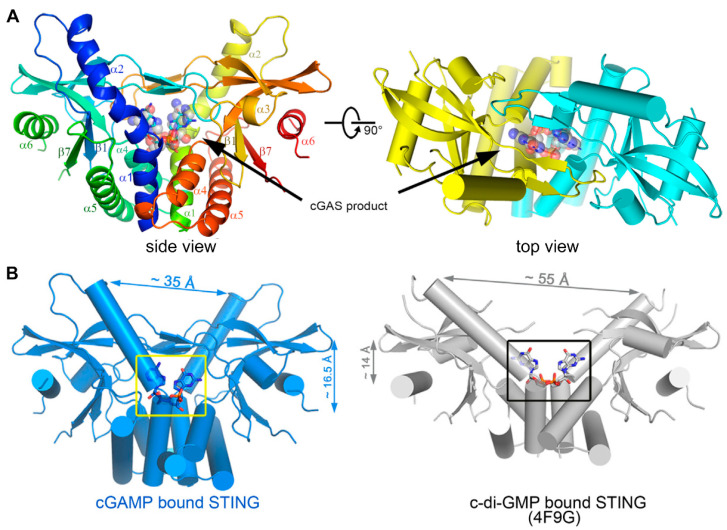
(**A**) Overall structure of STING C-terminal domain bound to the cGAS product. STING forms a dimer and is colored in yellow and cyan for each molecule, respectively, in the right panel, and the 2′3′-cGAMP molecule is indicated. (**B**) A detailed view of the cGAS product within the STING structure. cGAMP binding at a deeper pocket drags the STING dimers (blue) closer to each other, compared to c-di-GMP-bound STING (PDB ID code 4F9G, gray). Structures are adapted with permission from Ref. [53]. Copyright 2013, Elsevier Inc.

Cyclic dinucleotides (CDNs) consist of cyclic di-GMP (c-di-GMP), cyclic di-AMP (c-di-AMP), and cGAMP. After activation by DNA, cGAS catalyzes the synthesis of cGAMP, which then activates the STING-mediated IFN-I response [46]. cGAMP has canonical or noncanonical structures according to the orientation of phosphodiester bonds. Canonical CDNs with a 3′,3′ linkage orientation originate from bacteria; however, they have been largely disregarded as potential drug candidates for their inability to activate all human STING (hSTING) isoforms and translational limitations [47]. Noncanonical CDNs include synthetic forms like 2′,2′-cGAMP, which has two 2′,5′ phosphodiester bonds that do not exist naturally [48,49]; 3′,2′-cGAMP, which is identified in Drosophila melanogaster as a product of cGAS-like receptors to recognize dsRNA [50,51]; and 2′,3′-cGAMP, which is generated intracellularly by mammalian cGAS and shows affinity for hSTING variants. Their affinity for hSTING is in the order of 2′,3′-cGAMP > 2′,2′-cGAMP > 3′,3′-cGAMP ≈ 3′,2′-cGAMP [52,53]. The binding profiles of 2′,3′-cGAMP to the STING C-terminal domain and a detailed view of cGAMP within STING are shown in Figure 3 [53]. Natural CDNs are poor drug candidates due to their susceptibility to hydrolytic degradation by phosphodiesterases; but they could provide insights into the design of STING agonists.

**Figure 4:**
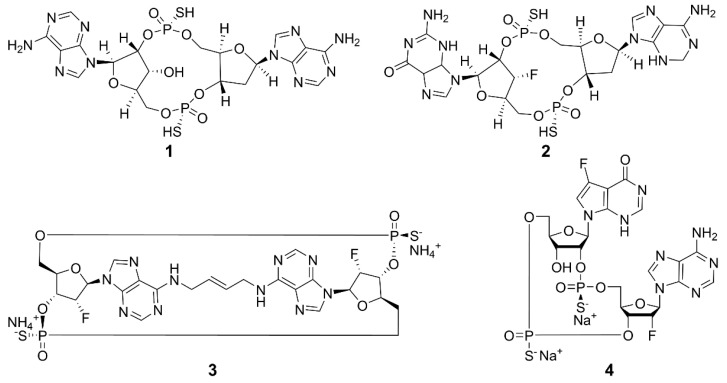
Structures of CDN-type STING agonists.

Current research is exploring chemically modified CDNs and non-nucleotide small molecules as synthetic agonists for hSTING. The emphasis is on synthetic CDN analogs that are nonhydrolyzable and contain a combination of 2′,5′ and 3′,5′ phosphorothioate bonds, mimicking the structural characteristics of natural CDNs [54], such as 3′,3′-cGAMP and 3′,2′-cGAMP. The structures of some examples are shown in Figure 4. Compound **1** (ADU-S100) is the first CDN class STING agonist in clinical trials, which has exhibited high affinity for both mouse STING (mSTING) and hSTING, and significantly enhanced the production of IFN-I. The mixed phosphorothioate bonds improve the stability and lipophilicity. The substitution of nonbridging oxygen atoms at the phosphate bridge with sulfur atoms enhances resistance to phosphodiesterase degradation, potentially promoting cellular uptake and cytosolic delivery [55,56,57]. Despite its favorable features, the clinical trial of **1** for recurrent or metastatic squamous cell carcinoma was terminated due to limited antitumor efficacy [58]. Compound **2** (MK-1454) strongly activated STING in head and neck squamous cell carcinoma and has entered phase II clinical trials [59]. Compound **3** (E7766) demonstrated promising antitumor effects in mice, including the ability to induce tumor-specific immune memory. In order to improve the therapeutic efficacy, transannular macrocyclic bridges were used to stabilize the CDN in its bioactive U conformation [60]. Other agonists, such as **4** (TAK-676), have entered phase I clinical trials to investigate antitumor effects [61]. The synthesis of natural CDNs and their analogs is a precondition for the clinical application of CDN agonists. In this regard, several challenges need to be addressed. For instance, modified CDNs often exhibit poor metabolic stability, low cellular uptake, and limited utilization rates [62]; most clinical studies rely on intra-tumoral drug delivery, and the large-scale industrial synthesis of CDNs remains limited. These challenges may be overcome by the rational design and synthesis of novel CDN analogs, the development of efficient delivery systems, and the application of combination therapies.

### 2.2. Non-CDNs

**Figure 5:**
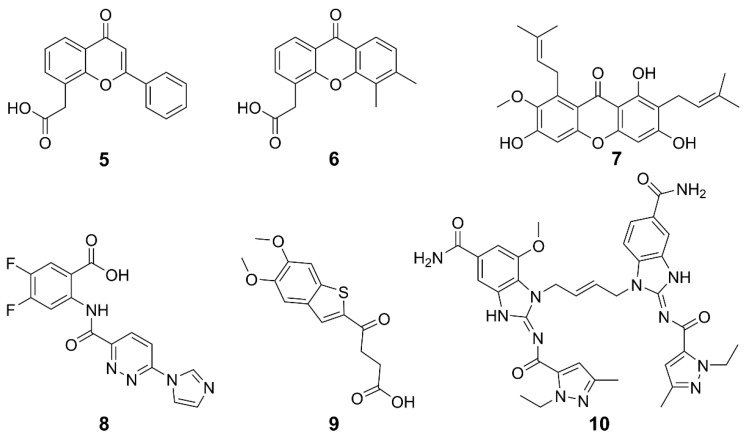
Structures of non-CDN-type STING agonists.

Non-CDN-based agonists for STING have been developed as promising alternatives to CDNs. STING can be activated by multiple non-CDN agonists, such as synthetic small molecules, natural products, and so on. The structures and general information of some STING agonists are shown in Figure 5 and Table 1. Flavone acetic acid (FAA) **5** was initially utilized as a vascular-disrupting agent and exhibited therapeutic potential by inducing hemorrhagic necrosis in murine tumors [34,63]. The exploration of STING agonists for cancer treatment commenced with the investigation of **5**, predating the discovery of the STING pathway; however, it was hindered by a narrow therapeutic window and poor pharmacokinetic properties [64]. To meet these challenges, a refined derivative **6** (DMXAA) was developed [65,66]. Originally an anti-angiogenic drug, **6** displayed a potent cytotoxic effect on tumor cells across various mouse models. Subsequently, it was found that both **5** and **6** can directly interact with STING, activating its signaling cascade and thus displaying anticancer potential [67]. However, **6** failed in phase III clinical trials for non-small cell lung cancer, which was attributed to its inability to activate hSTING except mSTING [68,69], and to induce downstream IFN-I signaling [70,71,72]. The differences between hSTING and mSTING are quite subtle, suggesting that further fine modifications on the structure of **6** may lead to effective hSTING agonists [34]. In fact, some derivatives of **6** were designed to target the hSTING [73]. Compound 7 (α-Mangostin) is a weak agonist of hSTING and a natural product isolated from mangosteen, possessing the same anthrone skeleton as **6** [73,74]. It showed higher efficiency in activating hSTING than in activating mSTING. These studies lay the foundation for the rational design of **6**-like STING agonists. Species specificity remains a key challenge to be overcome. Therefore, the optimization of small molecules akin to **6** may be a feasible strategy for discovering novel inducers of human interferon to treat cancers.

Compound **8** (SR-717) was screened from 100,000 compounds and its membrane permeability was optimized for systemic and oral administration. Crystallographic analysis revealed that two **8** bind at the base of the STING dimer inter-subunit cleft, inducing a closed-lid conformation resembling the binding mode of 2′,3′-cGAMP. The paired **8** interact with the same contact residues as 2′,3′-cGAMP, allowing them to competitively bind to STING. Intraperitoneal administered **8** at doses (15 or 30 mg kg^–1^) increased cytokine levels and exhibited significant antitumor activity against B16-F10 melanoma and MC38 colorectal adenocarcinoma in mice. Moreover, it hindered lung nodule formation, showing a potential against metastasis. Compound **8** binds to both hSTING and mSTING variants and has entered preclinical studies for melanoma [75].

Benzothiophene oxobutanoic acid **9** (MSA-2) was identified to be a potential STING agonist by high-throughput screening of a library containing about 2.4 million compounds [76]. It exhibited high cellular permeability and STING selectivity, and induced dose-dependent phosphorylation of both TBK1 and IRF3. Interestingly, it formed a noncovalent dimer in solution and bound to the CDN-binding pocket of STING with high affinity (KD = 8 nM) and slow off-rate (t_1/2_ = 1.3). The dimerized **9** interact through the aromatic cores, pre-associating in solution before binding to STING at the same site as 2′,3′-cGAMP. This binding mode stabilizes a closed-lid conformation similar to that induced by 2′,3′-cGAMP-bound STING, effectively bridging across the STING homodimer and noncovalently cross-linking the two subunits. The selectivity of **9** for tumor tissue was shown by the higher concentrations of **9** in subcutaneous MC38 tumors than in plasma or organs, which increased production of IFN-β and proinflammatory cytokines at the tumor site. Consequently, treatment with **9** led to complete regression of tumors in 80–100% of mice bearing MC38 colon carcinoma when administered intratumorally, subcutaneously, or orally. Remarkably, orally administered **9** in mice showed comparable or superior efficacy to a cGAMP analogue delivered intratumorally or subcutaneously, demonstrating its great clinical potential. In terms of immune modulation, **9** demonstrated robust long-term antitumor immunity and enhanced the efficacy of immune checkpoint blocker (ICB) to PD-1 in MC38 and CT26 colorectal, LL-2 lung cancer and B16-F10 melanoma models. In brief, **9** enhanced the selectivity of STING agonists, thus overcoming the unexpected systemic inflammation and associated toxicity resulting from excessive activation of the cGAS-STING pathway in normal tissues.

In 2018, the first non-CDN-type STING agonist **10** (di-ABZI) was reported, which induced a potent antitumor activity to completely curb the tumor [77]. This agonist was derived from the modification of amidobenzimidazole, which has a certain affinity for the STING subunit and dimerization further enhanced the affinity. Similar to cGAMP, **10** selectively induced dose-dependent activation of STING and secretion of IFN-β, exhibiting over 400-times greater potency than cGAMP in human peripheral blood mononuclear cells. Analysis on the binding mechanism revealed that cGAMP induced a closed conformation of the STING, while **10** bound to STING in an open conformation. The success of **10** suggests that STING agonists could be developed on a new mechanism of action. The evaluation on the anticancer effects of **10** in a syngeneic colon tumor model demonstrates that it induced tumor eradication in 80% of the treated mice and significantly improved the survival rates.

**Table 1:** Status of cGAS-STING agonists in clinical trials.

Compound	Subject Tumors	Adimistration	Combination	Phase	NCT Number	Reference
**1**	Recurrent or metastatic squamous cell carcinoma of the bronchus	i.t.^†^	Alone	II	NCT03937141	[58]
**2**	Head and neck squamous cell carcinoma	i.t.	Alone and combined with pembrolizumab	II	NCT04220866	[59]
	Solid tumors and lymphoma	i.t.	Alone and combined with pembrolizumab	I	NCT03010176	[58]
**3**	Advanced solid tumor; lymphoma	i.t.	Alone	I	NCT04144140	[60]
	Urinary bladder neoplasm	Intravesical injection	Alone	I	NCT04109092	
**4**	Solid neoplasm	i.v.	Alone and combined with pembrolizumab following radiotherapy	I	NCT04420884	[61]
	Carcinoma, non-small-cell lung, triple-negative breast neoplasm, squamous cell carcinoma of head and neck	i.v.	Alone and combined with pembrolizumab following radiotherapy	I	NCT04879849	
**5**	Various malignant solid tumors	i.v.	Constant infusion with alkalinization	/	/	[78]
**6**	Solid tumors	i.v.	Alone	I	NCT00863733	[79]
	Non-small cell lung cancer	n.s.	Alone and combined with carboplatin and paclitaxel	I/II	NCT00832494	
**7**	Cervical cancer stem-like cells	i.g.	Alone and combined with cisplatin	/	/	[80]
	Colon cancer stem cells	i.p.	Alone and combined with 5-FU			[81]
**8**	Melanoma	i.p.	/	Preclinical studies	/	[75]
**9**	Colorectal carcinoma	i.t., i.h.; p.o.	/	Preclinical studies	/	[76]
**10**	Colorectal carcinoma	i.p.	/	Preclinical studies	/	[77]

^†^ i.t., intratumoral injection; i.v., intravenous injection; n.s., not specified; i.g., oral gavage; i.p., intraperitoneal injection; i.h., subcutaneous injection; p.o., oral administration.

### 2.3. Metal-Based cGAS-STING Agonists

Some metallodrugs could participate in both innate and adaptive immunity as chemoimmunotherapeutic agents to reverse immune evasion [82]. Recent studies show that metal complexes are promising agonists of the cGAS-STING pathway. Platinum drugs are the first-line chemotherapeutic agents in clinics for most solid tumors. They work by damaging DNA in tumor cells, leading to the production of DNA fragments and activation of the cGAS-STING pathway. Combining cisplatin and STING agonists can synergistically activate the cGAS-STING pathway, implying a close relationship between Pt complexes and this pathway [83]. For example, Pt^II^ triphenylamine complexes **11** and **12** could damage mitochondrial/nuclear DNA and the nuclear envelope to activate the cGAS-STING pathway, inducing pyroptosis in cancer cells and an intense anticancer immune response in vitro and in vivo [84]. A novel Pt^IV^ complex **13** is composed of oxaliplatin and acetaminophen; it induced STING activation and mitochondrial membrane remodeling, leading to pyroptosis [85]. Additionally, two Pt^IV^ complexes **14** and **15** bearing MSA-2 demonstrated significant antitumor immune effects and stimulation on the STING pathway. This strategy suggests that combining DNA-damaging agents with STING agonists is a promising approach for cancer therapy [86].

**Figure 6:**
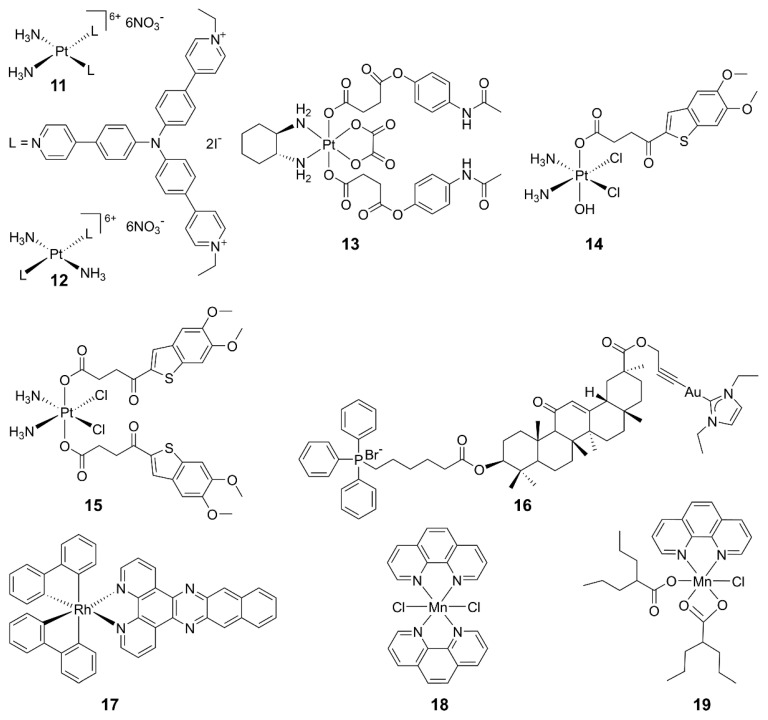
Structures of representative metal complexes as cGAS-STING agonists.

Other metal complexes have also shown promising regulatory effects on the cGAS-STING pathway. For example, mitochondria-targeted gold(I) complex **18** was designed, which generated a substantial amount of reactive oxygen species (ROS) and facilitated DNA excretion. The ROS-induced immunogenic cell death (ICD) and the DNA-activated cGAS-STING pathway triggered a robust anticancer immune response in hepatocellular carcinoma. This approach may offer an innovative strategy for designing chemoimmunotherapeutics [87]. The impact of mitochondrial DNA (mtDNA) on cancer immunotherapy has come into view. A rhodium(III) complex **19** has been reported as an mtDNA intercalator, inducing the release of mtDNA fragments into the cytoplasm and activating the cGAS-STING pathway. This study provides valuable insights into the development of biomacromolecule-targeted immunotherapeutic agents [88]. The structures of some representative metal-based cGAS-STING agonists are shown in Figure 6.

In 2018, Jiang discovered that viral infections lead to a significant increase in the cytoplasmic concentration of Mn^2+^, and there was a close dependency between Mn^2+^ and the cGAS-STING pathway [89], which has aroused the interest in Mn^2+^ for immunotherapy. Mn^2+^ has three main effects on the cGAS-STING pathway: direct activating cGAS and inducing a noncanonical catalytic synthesis of 2′,3′-cGAMP [89,90]; enhancing the binding ability of STING to cGAMP, thereby strengthening the cGAS-STING pathway [89]; and activating cGAS in a non-dsDNA-dependent manner [91,92]. Currently, several tumor treatment strategies based on the activation of the cGAS-STING pathway by Mn^2+^ have been developed [92]. Mn^2+^ was demonstrated to activate the STING pathway in colon cancer cells, leading to production of IFN-I. It downregulated dihydroxyacid dehydratase expression in tumor cells, inducing lipid peroxidation and increasing ROS levels, ultimately triggering ferroptosis [93].

While Mn^2+^ monotherapy shows promise, combination therapies leveraging the immunomodulatory effect of Mn^2+^ and other drugs may bring more encouraging results. An anthracycline antitumor drug doxorubicin (DOX) was encapsulated in amorphous porous manganese phosphate (APMP) nanoparticles with a phospholipid (PL) outer layer, forming PL/APMP-DOX nanoparticles [94]. The PL layer protects the nanoparticles and targets tumor cells. In the acidic TME, these nanoparticles degraded, rapidly releasing Mn^2+^ and DOX to activate the cGAS-STING pathway. Moreover, biomineralized manganese oxide nanoparticles (Bio-MnO_2_ NPs) were reported to convert endogenous H_2_O_2_ to O_2_, enhancing the radiosensitivity of non-small cell lung cancer cells [95]. The release of Mn^2+^ from these nanoparticles activates the STING pathway, significantly inhibiting tumor growth in mice with lung cancer [96].

**Figure 7:**
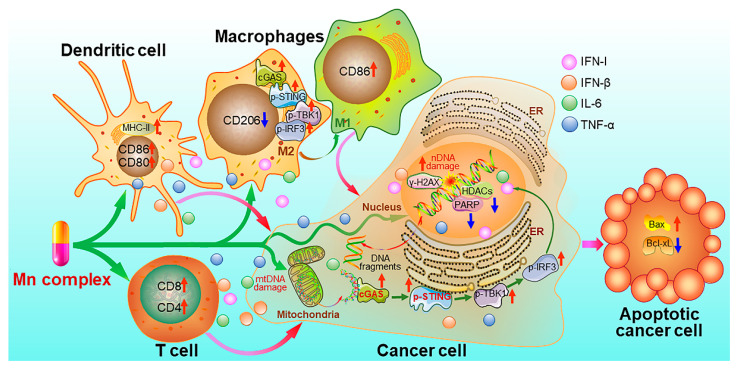
Proposed mechanism of action for manganese complexes **16** and **17** [97].

Recently, we synthesized Mn^II^ complexes **16** and **17** (MnPC and MnPVA), where 1,10-phenanthroline is a non-toxic DNA intercalator, and valproic acid is an inhibitor of histone deacetylases (HADCs). They not only caused DNA damage but also inhibited its repair, leading to the leakage of DNA fragments into the cytoplasm to activate the cGAS-STING pathway in tumor and immune cells. As a result, tumor infiltration by DCs and macrophages was enhanced, IFNs and pro-inflammatory cytokines were increased, and cytotoxic T cells were stimulated to kill cancer cells both in vitro and in vivo. Notably, **16** and **17** exhibited greater immunocompetence and antitumor activity than Mn^2+^ ions, demonstrating their potential as promising chemoimmunotherapeutic agents for cancer treatment [97]. They are the first multifunctional Mn^II^ complexes that suppress tumor cells, mainly through activating antitumor immunity via the DNA damage-initiated cGAS-STING pathway. Their anticancer mechanisms are show in Figure 7.

Although metal-based cGAS-STING agonists have demonstrated promising activities, the clinical application of these compounds is limited by their side effects [98]. For example, Pt drugs exhibit poor selectivity, affecting not only cancer cells but also healthy tissues, leading to severe side effects [99]. Additionally, hematologic toxicity, hepatotoxicity, cardiotoxicity, gastrointestinal toxicity, and hypersensitivity reactions involving immune reactions can pose serious problems [100,101,102]. The risk of secondary immune-related cancers following metal drug therapy still exists [98]. In short, a deep understanding of the mechanisms of metal complexes is needed for the design of more efficient and less toxic cGAS-STING agonists.

### 2.4. Other Agonists

β-Arrestin 2 plays an important role in numerous signaling cascades associated with various diseases, such as metabolic disorders and cancers, primarily through G protein-coupled receptor (GPCR) pathways [103]. Recent studies revealed that β-arrestin 2 interacts with cGAS, promoting the binding of dsDNA to cGAS and subsequently enhancing the production of cGAMP. This interaction leads to the deacetylation of β-arrestin 2 at Lys171, facilitating the activation of the cGAS-STING pathway and the production of IFN-β, ultimately contributing to immune evasion [104].

Chitosan is an appealing alternative of alum in vaccine adjuvants due to its composition of N-acetylated and deacetylated glucosamines [105]. It can trigger the release of intracellular DNA, activating cGAS and initiating the cGAS-STING pathway, and thereby promoting the production of IFN and IFN-stimulated genes (ISGs) [106]. Chitosan-based adjuvants are suitable for oral and intranasal vaccines and are gaining recognition for their cost-effectiveness, easy availability, high biocompatibility, and biodegradability. However, the limited water solubility restricts their application. Additionally, several protein agonists have been identified to activate cGAS [106]. For instance, GTPase-activating protein SH3 domain-binding protein 1 (G3BP1) enhances DNA-binding affinity [106,107], tripartite motif-containing 21 (TRIM21) aids in cGAS detection in viral genomes [108], zinc-finger CCHC-type-containing protein 3 (ZCCHC3) enhances cGAS sensing of DNA and oligomerization [109], and poly(rC)-binding protein 1 (PCBP1) enhances cGAS sensing of DNA and oligomerization [110].

In brief, despite the structural and source diversity, indirect agonists of the cGAS-STING pathway mainly function through promoting intracellular ROS production, transcriptional regulation, and ectonucleotide pyrophosphatase phosphodiesterase 1 (ENPP1) inhibition. These mechanisms provide enlightenment for the development of cGAS-STING pathway agonists.

## 3. cGAS-STING Inhibitors

**Figure 8:**
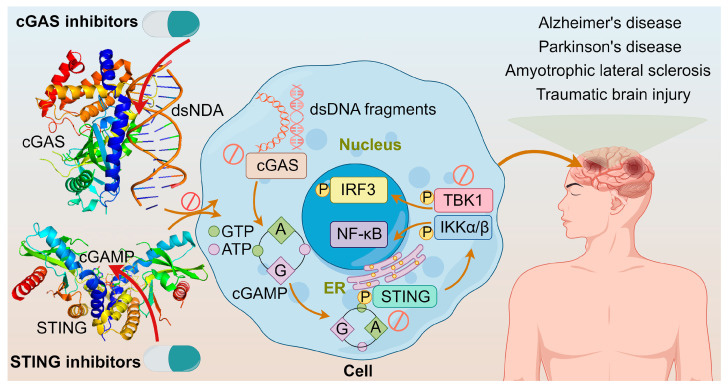
The crystal structures of cGAS dimer with dsDNA (PDB ID: 6ct9) and STING with 2′,3′-cGAMP (PDB ID: 4ef4); cGAS inhibitors directly inhibit cGAS or cGAS binding to dsDNA; STING inhibitors inhibit STING palmitoylation, CDN pocket, or direct transplanting of TBK1; and diseases associated with the overactivation of cGAS-STING pathway.

As understanding on the cGAS-STING pathway deepens, it is realized that excessive activation of this pathway could prolong the expression and secretion of inflammatory cytokines like IFN, leading to autoimmune diseases, such as Aicardi–Goutieres syndrome and lupus [111,112]. The diseases are triggered mainly by two ways: one involves abnormal accumulation of DNA, leading to continuous activation of the cGAS-STING pathway and sustained release of inflammatory cytokines like IFN-I; the other involves mutations in STING itself, causing its continuous activation independent of upstream signals, thereby leading to autoimmune diseases [113]. Furthermore, neurological disorders such as Alzheimer’s disease, Parkinson’s disease, amyotrophic lateral sclerosis, traumatic brain injury, and age-related diseases are closely associated with abnormal activation of this pathway [114]. These adverse effects constitute the alarm system of cGAS-STING activation. Modulating the cGAS–STING pathway may offer a new approach to treating acute or chronic inflammatory diseases shown in Figure 8. It was demonstrated that cGAS activation causes autoimmune diseases in Trex1^-/-^ and DNaseII^-/-^ mice, suggesting that inhibiting cGAS could prevent and treat some human autoimmune diseases triggered by self-DNA [115]; and tonic prime-boost of STING signaling mediates Niemann–Pick disease type C [116]. These examples rationalize the development of cGAS-STING inhibitors.

### 3.1. STING Inhibitors

**Figure 9:**
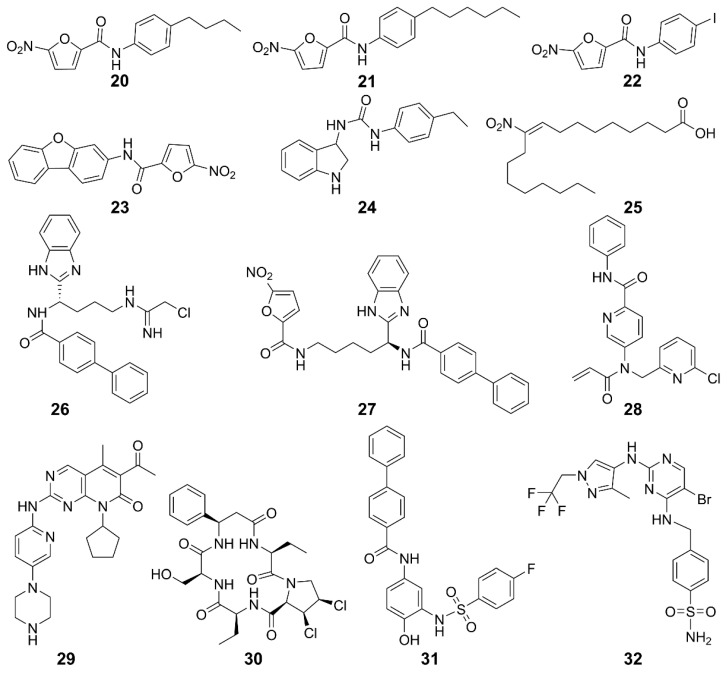
Structures of STING inhibitors.

STING inhibitors hold great potential for treating tumors and inflammatory diseases, warranting further investigation in clinical trials [117,118]. Based on the inhibition mode, they can be categorized into palmitoylation inhibitors, CDN pocket inhibitors, and TBK1 inhibitors. Some of these inhibitors and their structures are summarized in Figure 9 and Table 2.

The STING activity is affected by polyubiquitination and phosphorylation, sumoylation, nitro-alkylation, oxidation, carbonylation, and disulfide bond formation [117,119]. Blocking STING palmitoylation represents an effective strategy for inhibiting the STING activation. STING inhibitors disrupt palmitoylation by covalently binding to Cys88 or Cys91 residues in the transmembrane region of the STING protein [120]. Nitrofuran derivatives **20**–**23** effectively inhibited STING palmitoylation and strongly reduced the STING-mediated IFN-β reporter activity [120]. Similarly, **24** inhibited STING palmitoylation through irreversible Cys91-dependent modification with higher specificity for hSTING over mSTING, thus regulating systemic inflammation [120]. Additionally, endogenous nitro-fatty acid **25** mediates anti-inflammatory, antioxidant, and cytoprotective effects. Their derivatives also covalently modified the Cys88 and Cys91 residues of STING to inhibit its palmitoylation, thereby inhibiting the production of IFN-I in fibroblasts from patients with STING-associated vasculopathy in infancy [121]. Compound **26** and its analog **27** inhibited STING oligomerization and activation by covalently modifying Cys148 [122,123]. Compound **28** formed adducts with Cys91 of STING, as well as with cysteines of other immune-related proteins [124]. Furthermore, it inhibited cGAMP-mediated STING in peripheral blood mononuclear cells. The cyclin-dependent protein kinase (CDK) inhibitor **29** (palbociclib) directly binds to STING and inhibits its activation in both mouse and human cells. Mechanistically, **29** targets Y167 of STING to block its dimerization, binding with cyclic dinucleotides and trafficking [125].

CDN pocket inhibitors target the C-terminal ligand-binding domain of STING, competitively binding to STING endogenous ligands. Furthermore, they promote the degradation of STING protein and regulate the STING pathway negatively. The natural cyclic peptide **30** was derived from the purple periwinkle plant [120]. It binds specifically to the activation pocket of the STING C-terminal, preventing the recruitment of IRF3 and maintaining the integrity of the STING-TBK1 interaction, thereby inhibiting the activation of the downstream signaling pathway mediated by STING [120]. It exhibited low cytotoxic side effects in mouse models and was expected to be improved after further modifications [126]. Compound **31**, as a specific STING inhibitor that binds to the CDN-binding pocket, is one of the promising lead compounds for the treatment of diseases related to aberrant STING activation [121]. It was discovered to compete with cGAMP for STING binding in the computer docking, with the affinity for the STING CDN-binding pocket being higher than cGAMP (IC_50_ = 76 nM). It locked STING into an open, inactive conformation, inhibiting its activation and thus the production of IFN-I and inflammatory cytokines in Trex1^-/-^ mice.

Inhibiting TBK1 is a viable method for STING inhibition. TBK1 inhibitor **32** is an ideal probe for the analysis of biological properties of TBK1 in immune, neuroinflammatory, obese, or cancerous models [127,128]. It inhibited Toll-like receptor 3-induced IFN-III phosphorylation in Ramos cells and IFN-I secretion in human primary monocytes. In monocyte-derived macrophages, **32** responded to dsDNA and cGAMP, inhibiting the secretion of IFN-β [129].

**Table 2:** STING inhibitors subjected to preclinical trials.

Function	Compound	Subject	Model	Activity	Reference
STING palmitoylation inhibitors	**20**, **21**	Human/Mice	Trex1^-/-^ mice	Improves systemic inflammation	[120]
	**22**, **23**	Mice	Trex1^-/-^ mice	Improves systemic inflammation	[120]
	**24**	Human/Mice	Trex1^-/-^ mice	Improves systemic inflammation	[120]
	**25**	Human/Mice	SAVI patients with fibroblast cells	Inhibits the production of IFN-I	[121]
	**26**	Human/Mice	BMDMs cell	Inhibits the activation of STING in mouse and human cells and increases the survival rate of mice	[123]
			Peripheral blood monocyte		
			Trexl^D18N/D18N^ mice of AGS		
	**27**	Human/Mice	Monocyte	Inhibits the covalent modification of STING and block the cGAS-STING signaling pathway	[130]
			BMDMs cell		
	**28**	Human	Peripheral blood mononuclear cells	Inhibits cGAMP-mediated STING	[124]
	**29**	Human/Mice	HEK293T	Lowers the incidence of autoimmune diseases	[125]
			Mouse peritoneal macrophages		
			THP1 cells		
CDN pocket inhibitors	**30**	Human/Mice	Trex1^-/-^ BMDMs cells	Inhibits the expression of IFN-I and pro-inflammatory cytokines and reduces self-inflammatory response	[121]
			Trex1^-/-^ mice		
	**31**	Human/Mice	Trex1^-/-^ mice	Improves immune system	[131]
TBK1 inhibitors	**32**	Human	Ramos cells	Inhibits Toll-like receptor 3-induced IFN-III phosphorylation	[127]
			Human primary monocytes	Inhibits IFN-I secretion	
			Monocyte-derived macrophages		

### 3.2. cGAS inhibitors

**Figure 10:**
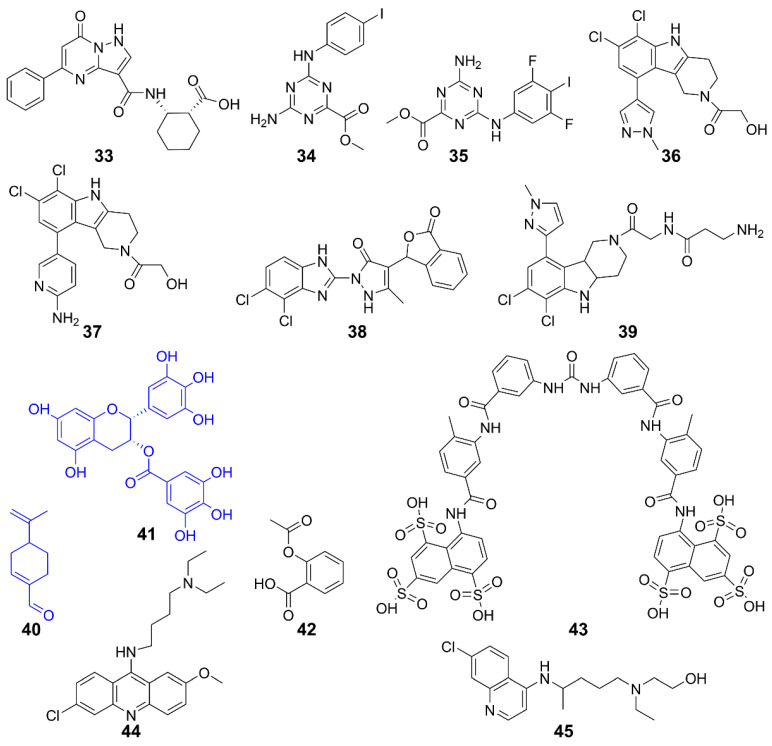
Structures of cGAS inhibitors.

Targeting cGAS is an important strategy for developing inhibitors of the cGAS-STING pathway. cGAS inhibitors include two categories—competitors for the ATP or GTP binding site of cGAS, and inhibitors of dsDNA binding to cGAS. Several such inhibitors are summarized in Figure 10 and Table 3.

A high-affinity inhibitor **33** was developed from a low-affinity fragment by Pfizer researchers. The biochemical and structural data suggest that it binds to the cGAS active site. Although its inhibitory effect at the cellular level was modest, it validated the druggability of cGAS inhibitors, provided a high-affinity tool compound, and established a high-throughput assay for identifying next-generation cGAS inhibitors [132]. Furthermore, **34** and **35** were identified as selective inhibitors of the DNA pathway in human cells, with no impact on the RIG-I-MAVS or Toll-like receptor pathways. These compounds introduced a novel class of hcGAS inhibitors that are active in cells, presenting a new chemical framework for creating probes to investigate cGAS function and develop treatments for autoimmune diseases [133]. Other hcGAS inhibitors such as **36** and **37** are optimized derivatives of the mcGAS inhibitor **38**. They occupy the catalytic site of cGAS, reducing its affinity for ATP and GTP and inhibiting its activity. The specificity and potency of these drug candidates were further demonstrated in human myeloid cells, including primary macrophages [134,135]. Further structural optimizations on both the side chain and central tricyclic core of **36** resulted in several subseries. Compound **39** is the most potent one with cellular IC_50_ values of 1.38 and 11.4 μM for h- and mcGAS, respectively. Mechanistic studies confirmed its direct targeting to cGAS. Furthermore, **39** exhibited superior in vivo anti-inflammatory effects in a lipopolysaccharide-induced mouse model [136]. Compounds **40** (perillaldehyde) exhibited strong inhibitory effects on both mcGAS and hcGAS and demonstrated excellent anti-inflammatory properties [137]. It inhibited cGAS activity, thereby suppressing innate immune responses triggered by cytosolic DNA. Consequently, the **40**-treated mice were more susceptible to herpes simplex virus type 1 (HSV-1) infection. Since **40** is derived from Perilla frutescens, the results suggest that natural compounds could be potential inhibitors of cGAS [136,137]. This speculation is further supported by natural compound **41** (epigallocatechin gallate, EGCG), which disrupted existing G3BP1-cGAS complexes and inhibited DNA-triggered cGAS activation [138,139]. Interestingly, **42** (Aspirin) was found to directly acetylate cGAS and efficiently inhibit cGAS-mediated immune responses [140]. Moreover, it effectively suppressed self-DNA-induced autoimmunity in Aicardi–Goutières syndrome (AGS) patient cells and in an AGS mouse model.

Disrupting the binding between cGAS and dsDNA is an approach to inhibit the activation of cGAS. Compound **43** (Suramin) was discovered to inhibit the STING pathway by displacing bound DNA from cGAS and inhibiting cGAS enzymatic activity. Compound **43** or its analogs could be utilized as anti-inflammatory drugs [141]. Compounds **44** (Acrinamin) and **45** (Oxychloroquine) specifically bind to the grooves of dsDNA at the interface of cGAS/dsDNA interaction, inhibiting the interaction between cGAS and dsDNA and thus suppressing cGAS activation [142]. These compounds may serve as potential therapeutics for diseases such as AGS or systemic lupus erythematosus (SLE).

**Table 3:** cGAS inhibitors in preclinical trials.

Function	Compound	Subject	Model	Activity	Reference
Inhibits cGAS	**33**	Human cGAS	/	/	[132]
	**34**, **35**	Human cGAS	THP-1 cells	Inhibits DNA pathway	[133]
	**36**, **37**	Human cGAS	THP-1 cells	Inhibits cGAS activity induced by dsDNA	[135]
			RAW 264.7 cells		
	**38**	Mice cGAS	AGS mice	Inhibits IFN-I expression	[134]
	**39**	Human/Mice cGAS	THP-1 cells	Inhibits inflammation	[136]
	**40**	Human/Mice cGAS	AGS mice	Improves self-induced inflammation response of DNA	[137]
	**41**	Mice cGAS	AGS mice	Suppresses DNA-triggered cGAS activation by preventing the formation of G3BP1-cGAS complex, reducing auto-inflammatory response	[139]
	**42**	Human/Mice cGAS	AGS patient cells and AGS mice	Inhibits cGAS-mediated immune responses and self-DNA-induced autoimmunity	[140]
Inhibits interaction with dsDNA/cGAS	**43**	Human cGAS	THP-1 cells	Inhibits cGAS-mediated IFN-I response	[141]
	**44**, **45**	Human/Mice cGAS	Trexl^-/-^ mice	Acts on AGS/SLE mice	[142]

## 4. Perspectives

The cGAS-STING pathway plays an important role in the pathogenesis and progression of cancers and autoimmune diseases. In most cases, activating this pathway leads to antitumor immune responses and cancer inhibition. Therefore, cGAS-STING agonists could be used alone or in combination with other drugs to treat various cancers. However, as a key inducer of IFN-I responses, cGAS-STING also promotes stage-specific tumor initiation and proliferation, and mediates the pathogenesis of autoimmune diseases. Thus, the side effects of STING agonists should be considered in cancer therapy. In this review, we have shown various approaches to produce modulators of the cGAS-STING pathway, nevertheless, several challenges remain to be addressed in the future.

Firstly, STING agonists face clinical challenges in tumor treatment because they show poor efficacy in cancer patients. For instance, ADU-S100 monotherapy demonstrated only a 2.1% overall response compared to untreated groups [143]. Similarly, MK-1454 showed no overall response for monotherapy when administered intratumorally, and a 24% overall response when combined with the anti-PD-1 monoclonal antibody pembrolizumab [144]. Neither of them consistently demonstrated abscopal effects with non-injected distal tumors shrinking. These results lowered the initial expectations on STING agonists usage as potential anticancer treatments. The results may also suggest that standard cancer therapy is insufficient, and combined therapies with chemotherapy, radiotherapy, or targeted therapy may produce more beneficial effects. The disparity between the preclinical efficacy and the initial clinical results further underscores the need to comprehensively study the biological and pharmacological mechanisms of STING agonists. A deep understanding of the mechanism can promote the development of new agents or drug combinations and allow us to fine-tune the inflammatory TME to fully exploit the potential of the cGAS-STING pathway in immunotherapy.

Secondly, CDNs like ADU-S100 are quickly cleared from injection sites (t_1/2_ = 10–20 min) due to their poor pharmacokinetics resulting from metabolic instability and membrane impermeability [145]. This limitation may be addressed by increasing the frequency of administration, but this poses challenges, especially for image-guided tumor injections. The transient drug exposure and localized STING activation at the tumor site may not be sufficient for generating the systemic T cell responses required for abscopal effects in in situ vaccination. Additionally, most STING agonists do not comply with the Lipinski’s rule—an empirical rule for evaluating the possibility of a compound to become a drug, leading to unsatisfactory clinical outcomes. They are also prone to degradation by phosphodiesterases and have limited modification possibilities due to large molecular weight. Therefore, it is necessary to design new compounds or drug delivery systems with improved pharmacokinetics. The aim is to improve cellular uptake, enhance cytosolic delivery, reprogram tumor-draining lymph nodes, and increase local retention of CDNs, which are highly important for intra-tumoral drug administration.

Thirdly, systemically administered STING agonists can induce a transient systemic inflammatory response resembling a cytokine storm, resulting in unexpected immune reactions and flu-like symptoms in patients. Particularly, T cells express high levels of STING and are vulnerable to STING-induced apoptosis [146,147]. Non-nucleotide small molecule STING agonists are potentially toxic to T cells [146]; they enter the cytosol directly through passive diffusion across cellular membranes, leading to uncontrolled STING activation in T cells and compromising antitumor adaptive immunity. A wide range of promising chemical, biomolecular, and pharmaceutical engineering approaches may solve this problem, with some being in development [148]. For instance, the non-nucleotide STING agonist MSA-2 utilizes a protonizable carboxylic acid group to enhance cellular membrane permeability in the acidic TME. Environmentally responsive drug carriers and conditionally cleavable chemical linkers have been developed, mainly for chemotherapeutics, to improve drug accumulation at tumor sites [149]. However, strategies to leverage TME for activating STING at tumor sites have not been extensively explored, which may enhance the effectiveness and safety of systemically administered STING agonists. The use of molecular targeting strategies, such as antibodies, peptides, and glycans, for STING agonists shows promise for achieving more tumor-selective activation of innate immunity and reducing inflammatory side effects.

Lastly, the use of cGAS-STING modulators may lead to excessive immune system activation and a cytokine storm, because cGAS and STING proteins are widely expressed across various tissues. In addition, systemic administration of STING agonists through intravenous or abdominal injection can overly activate the STING pathway, causing unnecessary inflammation in normal tissues. Therefore, cGAS-STING pathway modulators with tissue-targeting properties are required in the future to minimize the adverse reactions caused by the systemic immune effects. Novel drug delivery strategies such as antibody drug conjugates (ADCs) and drug release systems may help to avoid the adverse reactions of STING agonists.

## 5. Conclusions

The cGAS-STING pathway has emerged as a promising target in immuno-oncology. Academic researchers and pharmaceutical companies are actively developing agonists or inhibitors of the cGAS-STING pathway, aiming to translate them into applicable drugs. The importance of the cGAS-STING pathway for inflammation and immunity, coupled with checkpoint inhibitors, cytokine therapeutics, and cell-based therapies, underscore the compelling rationale for targeting this pathway in cancer immunotherapy. Preclinical studies of various STING agonists have shown encouraging results in tumor models, often leading to complete and long-lasting therapeutic responses in a majority of treated mice, even in models of highly immunosuppressive tumors [150]. Inhibitors of the cGAS-STING pathway are also potential drugs against autoimmune diseases and cancers. We believe that modulating the cGAS-STING pathway will become an effective strategy for the development of drugs to treat cancer and other autoimmune or inflammatory diseases in the future.

## Abbreviations

ABZI	Amidobenzimidazole
ADCs	Antibody-conjugated drugs
AGS	Aicardi–Goutières syndrome
APAP	Acetaminophen
APCs	Antigen-presenting cells
APMP	Amorphous porous manganese phosphate
CAFs	Cancer-associated fibroblasts
c-di-AMP	Cyclic di-AMP
c-di-GMP	Cyclic di-GMP
CDK	Cyclin-dependent protein kinase
cGAMP	Cyclic GMP-AMP
CTLs	Cytotoxic T-lymphocytes
Cys88/Cys91	Cysteine residue 88/91
DCs	Dendritic cells
DMBA	7,12-Dimethyl-benz(a)anthracene
DMXAA	5,6-Dimethylx-antheonone-4-acetic acid
DOX	Doxorubicin
dsDNA	Double-stranded DNA
EGCG	Epigallocatechin gallate
ENPP1	Ectonucleotide pyrophosphatase phosphodiesterase 1
ER	Endoplasmic reticulum
FAA	Flavone acetic acid
FIH	First-in-human
G3BP1	GTPase-activating protein SH3 domain–binding protein 1
GI	Gastrointestinal
GPCR	G protein-coupled receptor
GSH	Glutathione
hSTING	Human STING
HSV-1	Herpes simplex virus type 1
ICB	Immune checkpoint blockade
IDO	Indoleamine 2,3-dioxygenase
IFN-I	Type I interferon
IKK	IκB kinase
IL-6	Interleukin-6
IRF3	Interferon regulatory factor 3
ISGs	Interferon-stimulated genes
MDS	Myelodysplastic syndromes
MDSCs	Myeloid-derived suppressor cells
MHC-I	Major histocompatibility complex class I
mSTING	Mouse STING
NAFLD	Non-alcoholic fatty liver disease
NF-κB	Nuclear factor kappa B
NK	Natural killer
NP	Nanoparticle
Oxa	Oxaliplatin
PAH	Perillaldehyde
PAMPs	Pathogen-associated molecular patterns
PCBP1	Poly-binding protein 1
Pembro	Pembrolizumab
Phila	Cancer prev res
PL	Phospholipid
ROS	Reactive oxygen species
SAVI	STING-associated vasculopathy with onset in infancy
SLE	systemic lupus erythematosus
TBK1	TANK-binding kinase 1
TCGA	The Cancer Genome Atlas
TME	Tumor microenvironment
Tregs	Regulatory T cells
TRIM21	Tripartite motif-containing 21
ZCCHC3	Zinc-finger CCHC-type containing protein 3
ZF	Zinc-finger
